# Radiosurgery in Upper Tract Urothelial Carcinoma (UTUC)

**DOI:** 10.3390/cancers18060991

**Published:** 2026-03-19

**Authors:** Michael Staehler, Iulia Blajan, Can Aydogdu, Annabel Graser, Marina Hoffmann, Isabel Brinkmann, Gerald B. Schulz, Allen Yen, Jonathan J. Cheng, Christoph Fürweger, Sarah P. Psutka, Raquibul Hannan, Alexander Muacevic

**Affiliations:** 1 Department of Urology, University of Munich, D-81377 Munich, Germany; 2 European Radiosurgery Center Munich (ERCM), D-81377 München, Germanyalexander.muacevic@erc-munich.com (A.M.); 3 Department of Radiation Oncology, UT Southwestern Medical Center, Dallas, TX 75390, USAraquibul.hannan@utsouthwestern.edu (R.H.); 4 Department of Radiation Oncology, University of Washington, Seattle, WA 98195, USA; 5 Department of Urology, University of Washington, Seattle, WA 98195, USA; spsutka@uw.edu

**Keywords:** upper tract urothelial carcinoma, radiosurgery, hypofractionation, organ sparing treatment, urothelial carcinoma

## Abstract

**Objectives** Radiosurgery (RS) using high-dose hypo-fractionated radiation is a novel treatment for renal cell carcinoma. This study investigates long-term oncological outcomes of RS for upper tract urothelial carcinoma (UTUC) in patients unfit for surgery or with a solitary kidney. **Methods** We performed a retrospective analysis of three prospective institutional databases from tertiary referral centers, including patients with UTUC treated with RS between 2008 and 2024. **Results** The study included 45 patients with a median age of 71.9 years (range 47.8–89.1). Of these, 28 had high-grade disease, and 9 carcinoma in situ (CIS). Twenty-eight patients had a solitary kidney. The median RS dose per fraction was 25 Gy (range 10–25). The median number of fractions was 1 (range 1–4 fractions). The median follow-up was 27.6 months (range 3.1–150.3), and the median overall survival (OS) was 91.7 months (95% CI 72.0–111.5). OS did not correlate with tumor grade or CIS. The median OS for low-grade patients was 52.06 months, while for high-grade patients, it was 87.90 months. Treatment response included complete response in 37 patients (82.2%), partial response in 2 (4.4%), stable disease in 2 (4.4%), and progression in 2 (4.4%). The median baseline and follow-up CKD-EPI were 64.2 and 53.5 mL/min/1.73 m^2^, respectively. Three patients (6.7%) required hemodialysis. **Conclusions** RS for UTUC is an effective local ablation therapy with durable long-term control and should be considered in patients not suitable for surgical approaches. It should be evaluated as a potential new standard of care in prospective studies in conjunction with peri-interventional systemic therapy.

## Simple Summary

Upper tract urothelial carcinoma is an uncommon cancer often managed by removing the kidney and ureter. However, some patients cannot undergo major surgery or have just one working kidney, leaving them with few treatment options to avoid hemodialysis. This study examined whether a precise form of radiation therapy—mainly given in a single high-dose session—could safely control the disease while maintaining kidney function. Researchers reviewed long-term results from patients at specialist centers to evaluate survival rates, tumor response, and effects on kidney health. The results indicate that targeted radiation may offer lasting cancer control with manageable side effects, presenting a new possible alternative for those unable to have surgery and shaping future research into its place in routine care.

## 1. Introduction

Upper tract urothelial carcinoma (UTUC) is a rare condition that presents specific clinical management challenges and notable risks to patients, both from the disease itself and its treatments. It represents 5–10% of urothelial carcinomas, with an incidence in Western countries of approximately two cases per 100,000 inhabitants [1]. Although UTUC is often considered similar to urothelial carcinoma of the bladder, there are distinct pathogenic, genomic, biological, and clinical differences. For instance, UTUC of the renal pelvis is associated with a 5-year mortality rate exceeding 50%, which is significantly higher than the 25% rate for bladder cancer [1,2,3].

Prognosis is dependent on tumor-specific factors such as positive urine cytology, grading, location, multifocality, tumor diameter < 2 cm, pathological T stage, necrosis and treatment-related factors such as laparoscopic approach, extravesical bladder cuff removal, and positive surgical margins [4,5,6].

Treatment of UTUC poses specific challenges in this primarily older, often comorbid population, consisting primarily of perioperative systemic therapy and surgical removal of the kidney, ureter, and bladder cuff (radical nephroureterectomy, RNU), with nephron-sparing ablative approaches reserved for low-volume, low-grade disease [3,5,7,8,9,10]. Specifically, nephron-sparing approaches are also prioritized in patients in whom surgery is not an option or in those with a solitary kidney [3,5]. Patients with unresectable UTUC, including those ineligible for or refusing surgery (RNU or ureterectomy), are offered a clinical trial or best supportive care including palliative management of refractory symptoms like hematuria, which can involve radiation, systemic approaches, endoscopic, or ablative treatments [3]. Stereotactic body radiation therapy (SBRT) as a definitive treatment is currently not recommended or mentioned by the NCCN guidelines and has generally been underutilized in this setting [11]. SBRT has a specific advantage over surgery or ablative techniques in the management of UTUC: it is non-invasive, nephron-sparing, and may offer the preservation of renal function.

Since high-dose hypo-fractionated radiosurgery based on single-fraction approaches (RS) is now offered as a treatment option for renal carcinoma (RCC) and for patients with a functional or anatomical solitary kidney with UTUC [12,13], we aimed to describe the functional and oncological efficacy of this approach in a long-term cohort study involving multiple institutions.

## 2. Materials and Methods

Patients were retrospectively identified from prospectively maintained institutional radiosurgery databases at the participating centers. Inclusion criteria were harmonized across institutions and included histologically confirmed UTUC consecutively treated with RS between August 2008 and December 2024 in patients considered unsuitable for surgical treatment or in whom nephron preservation was imperative. Patients were considered unsuitable for surgical management based on multidisciplinary evaluation including comorbidity burden, advanced age, anesthetic risk, patient preference, or the presence of a solitary functional kidney where radical surgery would likely result in dialysis. All eligible patients treated with RS for UTUC during the study period were included in the analysis. Institutional Review Board ethics board approval was granted.

Tumor staging was based on pathological findings when available and otherwise on clinical staging derived from imaging and ureteroscopic evaluation. Intravesical or ureteral recurrences were classified as distant urothelial progression rather than local recurrence at the RS treatment site.

Response was assessed by a composite of Response Evaluation Criteria in Solid Tumors RECIST 1.1 on repeat MRI or CT imaging and ureterorenoscopy, including biopsies if indicated, and cytology every three months according to guideline recommendations. Kidney function was assessed using estimated glomerular filtration rate according to the chronic kidney disease formula (CKD-EPI). Adverse events were reported according to the CTCAE Ver. 3.0.

No patients received systemic therapy in direct conjunction with RS treatment.

Statistical analysis was performed with SPSS 29. For baseline characteristics, the chi-squared test was used for binary and categorical variables and the Wilcoxon rank test for continuous variables. Kaplan–Meier methods were used to estimate survival curves, and group comparison was conducted by using log-rank tests. Overall survival was defined as the time from RS treatment to death from any cause. Cox regression models were used to estimate hazard ratios. All reported *p*-values are two-sided, with statistical significance set at *p* < 0.05.

## 3. Results

A total of 45 patients were included with a median age of 71.9 years (range 47.8–89.1). Of these, 39 were male and 28 had high-grade disease, with nine patients having CIS (see Table 1). Localization of UTUC was in the renal pelvis in 38, renal calices in three, and proximal ureter in four cases. A total of 28 patients had a solitary renal unit after contralateral RNU. Four patients received more than one RS therapy (see Table 1).

**Figure 1:**
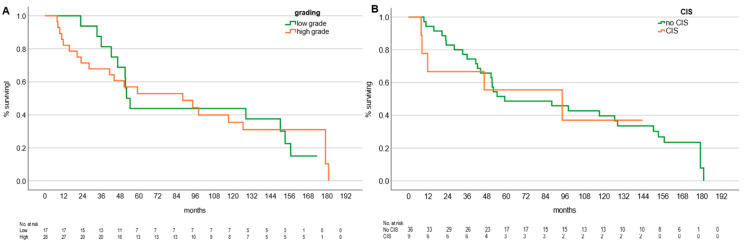
(**A**) Kaplan–Meier overall survival compared between patients with low- vs. high-grade UTUC. Median overall survival for low-grade patients was 52.1 months (95% CI 46.3–57.9), and 87.90 months (95% CI 22.2–153.7) for patients with high-grade UTUC; *p* = n.s.; (**B**) Kaplan–Meier overall survival comparing patients with/without CIS present or not. Median overall survival for patients without was 59.03 months (95% CI 5.6–112.5) and 94.3 months (95% CI 0.0–189.1) for patients with CIS; *p* = n.s.

**Figure 2:**
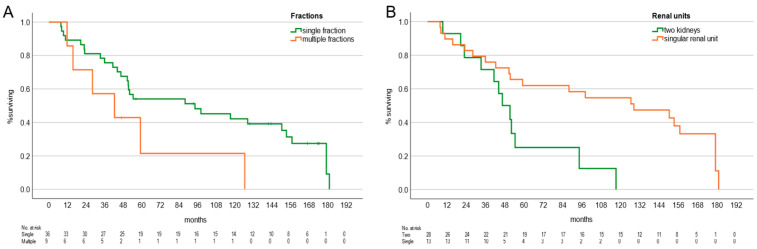
(**A**) Single vs. multiple fraction RS; OS Median OS in patients after single-fraction RS was 94.3 months (95% CI 21.8–166.7) vs. 42.3 months (95% CI 5.0–78.8) with multiple fractions; *p* = n.s.; (**B**) Kaplan–Meier overall survival stratified by number of renal units. Median overall survival for patients with two kidneys was 46.4 months (95% CI 34.3–58.3) and 128.27 months (95% CI 54.8– 201.7) for patients with a singular renal unit. Patients with bilateral at the start of SRS disease were omitted (n = 4); *p* = n.s.

The median RS dose per fraction was 25 Gy to the 70% isodose (range 10–25). The median fractions per patient were one (range 1–4 fractions). Median follow-up was 27.6 months (range 3.1–150.3). Median overall survival was 91.7 months (95% CI 72.0–111.5). High- vs. low-grade and presence vs. absence of CIS were not related to OS, with a median OS for low-grade patients of 52.06 months (95% CI 46.3–57.9) and 87.9 months (95% CI 22.2–153.7) for patients with high-grade UTUC (Figure 1A). Median overall survival for patients without CIS was 59.03 months (95% CI 5.6–112.5) and 94.3 months (95% CI 0.0–189.1) for patients with CIS (Figure 1B). There was a clinically meaningful but not statistically significant difference in median overall survival for patients with two kidneys at 46.4 months (95% CI 34.5–58.4) and 128.27 months (95% CI 54.8–201.7) for patients with a singular renal unit (Figure 2A). Median OS in patients after single-fraction RS was 94.26 months (95% CI 21.8–166.7) vs. 42.3 months (95% CI 5.9–78.8) with multiple fractions (Figure 2B).

Median glomerular filtration rate (CKD-EPI) at baseline was 64.2 mL/min/1.73 m^2^ (range 16.2–95.7) vs. 53.53 mL/min/1.73 m^2^ (range 10.5–85.1) at median follow-up, and three patients required hemodialysis.

**Figure 3:**
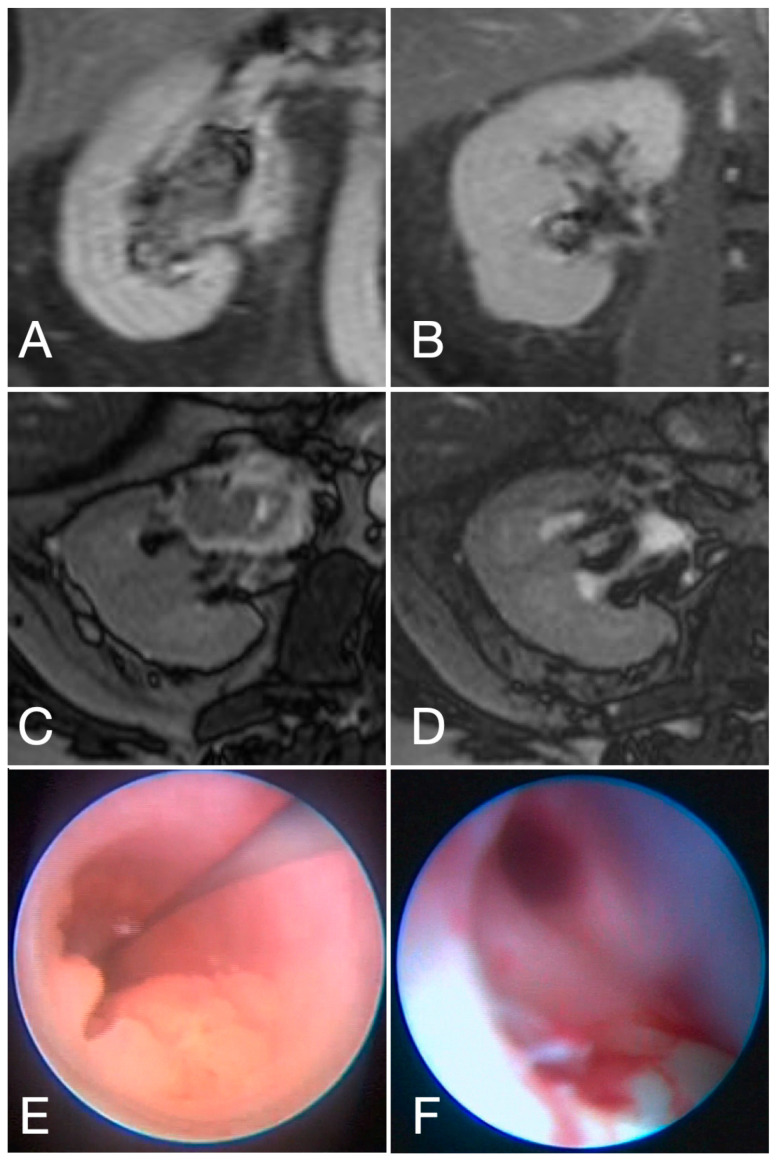
MRI and endoscopic results of UTUC at baseline (**A**,**C**,**E**) and 3 months post RS single-fraction therapy with 25GY to 70% percentile (**B**,**D**,**F**).

Composite response of the primary tumor estimated via URS (Figure 3) and MRI included complete response in 37, partial response in two patients, stable disease in two patients, and progressive disease in three patients with missing data in two cases.

A total of 12 patients experienced distant progression mainly within the bladder and ureter. N = 3 patients experienced distant metastasis, and n = 3 patients developed local progression at the site of prior treatment within a median of 21.56 months (range 1.9–58.4). In these three patients, n = 2 underwent RNU without histological proof of malignancy, although visible papillary structures were identified ureteroscopically. The remaining patient underwent RS directed at the recurrent disease, resulting in local response. Significant predictors of disease-free interval were singular renal unit (*p* = 0.04), singular vs. multiple fractions (*p* = 0.04), baseline creatinine (*p* = 0.036), and T-stage (*p* < 0.001). Tumor grade and CIS were not associated with survival (Figure 1).

Adverse events of RS were negligible with no long-term toxicity and only grade 1 fatigue within the first week after therapy in n = 17 patients.

## 4. Discussion

This is the first reported series of patients with UTUC treated with RS, demonstrating the potential of this treatment approach to support local tumor control and long-term survival, while preserving renal function and avoiding the need for renal replacement therapy in patients not suitable for surgery or at risk of dialysis after RNU.

We demonstrate that RS in UTUC can be performed safely and leads to an overall survival rate with a median of almost 5 years. Comparable data for this specific group of patients who are not surgical candidates are currently unavailable. Tumor control, both local and distant, was effective, and renal function was maintained with only three patients requiring dialysis. Baseline renal function demonstrated a moderate decline at follow-up (median 64.19 vs. 53.53 mL/min/1.73 m^2^), indicating that RS preserves renal function in most patients.

Currently, the only imperative indications for offering nephron-sparing treatment of UTUC include anatomically or functionally solitary kidneys, significant renal insufficiency with the imminent threat of hemodialysis, or bilateral disease [3,11]. Clinical experience with invasive UTUC patients on dialysis demonstrates survival of less than two years compared to approximately five years in patients with low-grade disease [14,15,16,17]. Thus, there is a paradigm shift towards nephron-sparing management [18]. Unfortunately, there are no specific recommendations on how to approach UTUC in singular kidneys [11].

In non-surgical candidates, contemporary guidelines predominantly recommend palliation for the management of symptoms such as gross hematuria, with radiotherapy being one of the potential palliative treatment options [3].

RS has been newly introduced as an alternative to surgical and ablative therapies for RCC by our team. Data and long-term follow-up experience have supported RS as a viable standard of care for patients with small renal masses (SRM < 4 cm) who are either unfit or unwilling to undergo surgery [12,19,20,21]. These efforts have led to RS being recommended for SRM in recent guidelines from the French Urologic Association (AFU) and the European Society of Medical Oncology [22,23,24]. The Guidelines for RCC of the European Association of Urology at least mention RS as a promising therapeutic option.

Through our collaboration with the International Radiosurgery Oncology Consortium for Kidney (iROCK), we have demonstrated both the long-term efficacy and the comparative effectiveness of single-fraction RS vs. fractionated RS for RCC [20,21].

RS for RCC may be risky when using traditional SBRT techniques, due to limited RS equipment and knowledge. Renal function deteriorates quickly by up to 41% with radiation exposure [14,25,26].

Effective treatment of RCC necessitates hypo-fractionated high doses of radiation alongside precise and narrow margins. It is important to clarify that RS should not be confused with SBRT, as RS continuously maintains motion in three dimensions through live tracking of body movement.

In our series, it was not possible to correlate tumor size and treatment size, as contouring was done not on diameters of the tumor but based on location. If the whole collecting system needed RS the number of fractions was increased to 3x at 12.5 Gy/fraction and to 4x at 10.0 Gy/fraction in a large kidney with chronic grade 3 dilatation.

The observed median OS of 91.7 months indicates favorable long-term outcomes, particularly in patients with a singular renal unit (128.27 months vs. 46.43 months in two kidneys, *p* = 0.04). This survival advantage may be attributed to the careful selection of patients with solitary renal units for RS, as well as the protective effect of preserving renal function. The high complete response rate (n = 37) further underscores the efficacy of RS, particularly when combined with URS and MRI for response evaluation. The low rates of local progression (n = 3) and distant metastases (n = 3) suggest that RS achieves durable local control, which is critical for patients with limited therapeutic options. In a recent report on 16 mainly elderly patients (median age 85 years) who underwent conventional SBRT with 40 Gy in 8 fractions every second day, only two patients had a complete response and two had disease progression within the treatment field [27]. Thus, hypofractionation with single-fraction RS seems to yield superior outcomes to conventional fractionation with SBRT, which was also shown in RCC [28,29].

It is important to note that OS for patients with two kidneys in our series was lower compared to those with a single kidney. Although patients with a single kidney were, on average, 5 years older and had a 9-month shorter follow-up, no other predictive factors could be identified to explain this effect. It is possible that patients with two kidneys may have had a higher likelihood of developing UTUC in the other kidney. When comparing these two groups it should be noted that patients with a single kidney after RNU represent a highly selected subgroup. These patients have already survived the initial UTUC and surgery and are likely under longer-term follow-up.

Our study population is selected towards patients not suitable for surgery; those with two kidneys presumably are patients in reduced physical and medical condition, exacerbating the effect of shorter survival expectations. Thus, the single renal unit group represents the more favorable subset of patients. However, subgroup analysis in small sample sizes may not be meaningful, given the observational nature of the study.

In RS commonly utilized in the patients reported here, fiducials must be inserted into the kidney, which is an invasive procedure. Initially, we attempted to position them within a secondary double-j stent in the renal pelvis, avoiding percutaneous insertion into the kidney. However, we abandoned this method due to unpredictable stent movement, rendering the planning unreliable. The fixed intracorporal fiducials; however, provide valuable information on renal movement.

Experience from using breathing commands while placing fiducials and conducting renal biopsies suggests that patients are unable to completely immobilize the kidneys despite halting thoracic breathing—they continue to rely heavily on diaphragmatic breathing, causing substantial kidney movement (up to 7cm; data on file). No other modalities offer real-time 3D tracking during therapy, except for the Cyberknife^®^ system (Accuray) and MRI-LINAC equipped with the Continuous Motion Management (CMM) upgrade using cine real-time MR imaging to monitor motion during radiation. MRI CMM is limited to real-time 2D imaging vs. 3D with the Cyberknife^®^ system. Nonetheless, this technology remains unsuitable for patients with even mild claustrophobia.

We observed that the impact of single-fraction RS on functionally relevant tissue is minimal, while the oncological effects, such as the rapid disappearance of the malignant tumor, are significant, as illustrated in Figure 3.

Median OS in patients treated with single-fraction RS was longer than that in patients treated with multiple fractions. While this may suggest a higher efficacy of single-fraction therapy, it is important to note that factors influencing the choice of fractionation—such as tumor location, proximity to radiosensitive adjacent organs, and target size—were not considered, due to the small sample sizes of these subgroups. Notably, we have seen the same effect in RCC, rendering single-fraction therapy the preferred and recommended approach [20].

Appropriate technology and a team consisting of urologic oncologists and radiation oncologists is needed for the successful execution of this new treatment paradigm. The main challenge in this setting is to clearly identify the tumor in the collecting system, and cross-sectional imaging is not reliable enough. Thus, the surgeon who saw the malignant lesions within the renal pelvis was part of the contouring and planning effort together with the radiation oncologist and radiation physicist.

Another key advantage of RS is that it can keep the ureter and renal pelvis functionally intact, as seen in our patients. This is a specific advantage of this approach over surgery or other ablative techniques for UTUC.

Late effect of radiation may lead to scar tissue forming and hyalinization which is a problem in other organs (i.e., kidney, lung, liver) and disrupts function, but for the upper urothelial tract RS does not disrupt functionality because the upper tract is mainly a conduit of the urine and scarring/fibrosis does not affect it as long as it is not leading to a stricture. As noted, hypofractionation at higher doses does not seem to have the same impact on regular tissue than traditional fractionated approaches [14].

The risk of renal functional loss and its associated patient morbidity places patients at a clinical disadvantage, necessitating specialized approaches and instrumentation for disease assessment, clinical staging, and management. Such aspects highlight the clear need for well-designed, multi-disciplinary strategies to guide optimal management for this vulnerable patient population to control variability and reduce the risks from under- and over-treatment. Emerging data from standardized paradigms for evaluation, counseling, and management provide a basis for appropriate risk-stratified approaches to optimize patient care, limit toxicity, and improve cancer control and survival. Curation and dissemination of this information, especially in a rare disease prone to clinical complexity, is critical to well-informed patient care and the consideration for referral to experienced, multi-disciplinary teams in more challenging cases.

### Limitations

Our study is limited by the relatively small sample size, limited follow-up, a selection bias, and a retrospective design of a non-formalized approach in a very rare cohort of the rare group of UTUC patients.

Within the highly selected group of patients who have a critical need to preserve renal function and lack additional local tumor control options, there is potential. Our data indicate a highly effective new approach that warrants a formalized prospective investigation into this aggressive oncological scenario.

## 5. Conclusions

RS for UTUC is a highly effective local ablation therapy with excellent long-term outcomes and should be considered in patients not suitable for surgical approaches. It should be evaluated as a new standard of care in prospective settings in conjunction with peri-interventional systemic therapy.

## Figures and Tables

**Table 1:** Baseline characteristics.

	n = 45
Median age (range)	71.9 years (range 47.8–89.1)
Male (%)	39 (85%)
High-grade (%)	28 (62%)
Carcinoma in situ CIS (%)	9 (20%)
Prior radical nephroureterectomy (%)	28 (62%)
*Location*	
Renal pelvis (%)	38 (84%)
Renal calyx (%)	3 (6%)
Proximal ureter (%)	4 (9%)
*Stage*	
pTis only	2 (4.5%)
pTa	24 (53%)
pT1	15 (33%)
pT2	4 (9%)
pT3	1 (2.2%)
pTx	1 (2.2%)
Median glomerular filtration rate (CKD-EPI)	64.2 mL/min/1.73 m^2^ (range 16.2–95.7)

## Data Availability

Access is restricted due to applicable German legal and regulatory requirements governing data protection and the handling of sensitive research data. Consequently, the data cannot be shared through the journal’s open access repository. Any potential access requests must comply with the relevant German legal framework and may require approval from the responsible data-holding institution and relevant authorities and may be directed to the authors directly.

## Abbreviations

The following abbreviations are used in this manuscript:

UTUC	Upper Tract Urothelial Carcinoma
RS	Radiosurgery
RCC	Renal Cell Carcinoma
RNU	Radical Nephroureterectomy
SBRT	Stereotactic Body Radiation Therapy
SRM	Small Renal Mass
CIS	Carcinoma in Situ
OS	Overall Survival
CI	Confidence Interval
CKD-EPI	Chronic Kidney Disease Epidemiology Collaboration (equation for estimating glomerular filtration rate)
CTCAE	Common Terminology Criteria for Adverse Events
RECIST	Response Evaluation Criteria in Solid Tumors
MRI	Magnetic Resonance Imaging
CT	Computed Tomography
SPSS	Statistical Package for the Social Sciences
NCCN	National Comprehensive Cancer Network
AUA	American Urological Association
SUO	Society of Urologic Oncology
EAU	European Association of Urology
ESMO	European Society for Medical Oncology
AFU	French Association of Urology (Association Française d’Urologie)
SEER	Surveillance, Epidemiology, and End Results Program
IROCK	International Radiosurgery Oncology Consortium for Kidney
CMM	Continuous Motion Management
Gy	Gray (unit of radiation dose)
pT	Pathological Tumor Stage
URS	Ureterorenoscopy
SRS	Stereotactic Radiosurgery
FASTRACK II	Focal Ablative Stereotactic Radiotherapy for Renal Cancer Trial II
TROG	Trans-Tasman Radiation Oncology Group
n.s.	Not significant
vs.	Versus
